# The combined impact of LLINs, house screening, and pull-push technology for improved malaria control and livelihoods in rural Ethiopia: study protocol for household randomised controlled trial

**DOI:** 10.1186/s12889-022-12919-1

**Published:** 2022-05-10

**Authors:** Abebe Asale, Menale Kassie, Zewdu Abro, Bayu Enchalew, Aklilu Belay, Peter O. Sangoro, David P. Tchouassi, Clifford M. Mutero

**Affiliations:** 1 International Centre of Insect Physiology and Ecology, Addis Ababa, Ethiopia; 2 International Centre of Insect Physiology and Ecology, Nairobi, Kenya; 3 University of Pretoria Institute for Sustainable Malaria Control, School of Health Systems and Public Health, University of Pretoria, Pretoria, South Africa

**Keywords:** Malaria, Study protocol, Randomized control trial, House screening, Long-lasting insecticidal nets, Push pull technology, Vector control, Jabi Tehnan, Ethiopia

## Abstract

**Background:**

The combined application of long-lasting insecticidal nets (LLINs) and indoor residual spraying (IRS) are commonly used malaria interventions that target indoor Anopheles vectors. Recent studies on the effects of house screening (HS) and LLINs have demonstrated a reduction in indoor vector densities and malaria when the interventions are combined. In addition, complementary interventions are needed to curb co-occurring pest populations which pose menace to agricultural crop productivity and food security. However, interventions that impact malaria mainly centre on public health strategies, overlooking subtle but important component of agricultural measures. Addressing the coexisting risks of malaria and crop pests could contribute to improved livelihood of communities.

**Methods:**

A four-armed household, cluster-randomized, controlled study will be conducted to assess the combined impact of HS, LLINs and push-pull agricultural technology (PPT) against clinical malaria in children in Ethiopia. The unit of randomization will be the household, which includes a house and its occupants. A total of 838 households will be enrolled in this study. In this trial 246 households will receive LLINs and HS, 250 will receive LLINs, HS and PPT, 175 households will receive LLINs and PPT. The remaining 167 houses which receive LLINs only will be used as control. One child aged ≤14 years will be enrolled per household in each treatment and followed for clinical malaria using active case detection to estimate malaria incidence for two malaria transmission seasons.

**Discussion:**

Episodes of clinical malaria, density of indoor biting malaria vectors, sporozoite infection rate, improved crop infestation rate, crop yield gain, livestock productivity and cost effectiveness analysis will be the end points of this study. Socio-economic, social demographic, cost-effectiveness analysis will be conducted using qualitative and participatory methods to explore the acceptability of HS and PPT. Documenting the combined impact of LLINs, HS and PPT on the prevalence of clinical malaria and crop pest damage will be the first of its kind.

**Trial registration:**

Pan African Clinical Trials Registry, PACTR202006878245287. 24/06/2020. https://pactr.samrc.ac.za/TrialDisplay.aspx?TrialID=11101.

**Supplementary Information:**

The online version contains supplementary material available at 10.1186/s12889-022-12919-1.

## Background

Malaria continues to be a major health threat in Africa where 93 and 94% of the global cases and deaths, respectively, are reported [1]. Long-lasting insecticidal nets (LLINs), indoor residual spraying (IRS), rapid diagnostic tests (RDTs) and prompt disease treatment are key strategies being used in fighting the disease [2]. Despite the proven effectiveness of LLINs and IRS for malaria control, their effectiveness may be undermined by widespread occurrence of insecticide resistance in vector populations [3, 4]. Thus, we are interested to know the impact of house screening as a supplementary vector control tool, as search for innovative vector control expands to accelerate malaria elimination efforts [2].

House screening (HS), a promising supplementary vector control tool, is a valuable non-chemical strategy for preventing indoor biting by vectors, by acting as a physical barrier against entry into human dwellings [5–8]. HS could serve as a potential alternative to IRS, thereby reducing the dependence on chemical insecticides for malaria control. HS is also a cheaper alternative to IRS. Recent studies have demonstrated that house screening can significantly reduce the number of mosquitoes entering houses in The Gambia [9, 10], Tanzania [11] and Ethiopia [12, 13]. Despite this evidence, quantification of the large-scale impact of HS in different eco-epidemiological strata have been limited. A large-scale HS efficacy trial are being conducted across Africa in search of novel vector control intervention tools. These include the studies conducted in western [14, 15] and southern Africa [16]. The efficacy of any new intervention tool can be affected by several factors including malaria epidemiology, agro-ecology, and socio-cultural elements of the communities. Thus, any new tool should be evaluated against these parameters. In this study, we propose to conduct the evaluation of HS intervention in Ethiopia under different agro-ecology and malaria epidemiology settings. As part of the intervention, the doors, windows, and eves of the house will be screened with polyethylene material to assess the impact of HS intervention in northwest Ethiopia.

In addition to introducing HS for malaria vector control, we are interested to know the impact of providing farmers with agricultural technologies on productivity. This is because agriculture and health has always been interconnected in many ways. Increasing agricultural productivity improves the overall livelihoods of communities through improved health, nutrition, and income generation through sale of crops and livestock [17]. A study conducted in Uganda suggests that a 10% increase in overall household income would reduce malaria incidence by 35.6% [18]. In this trial, Push pull technology (PPT), a biological method used for maize pest control will be introduced to selected farmers to improve their maize productivity and improve animal feed. It is a novel technology in which a repellent intercrop and attractant trap plant is used simultaneously. The stem borers are repelled from the food crop and simultaneously attracted to a trap crop, which leads to minimal survival of the pests’ immature stages.

A combination of interventions is commonly evaluated to improve malaria outcomes. For instance, the joint implementation of LLINs and IRS were found to significantly reduce malaria incidence [19, 20], although no such effect was observed in other studies which assessed the combined effect of both [17, 21]. Few studies have evaluated the combined effect of LLINs and HS. We hypothesize that, HS can be considered as a preferable intervention to supplement with LLINs since its duration, cost and its effect on the environment is lower than IRS, which is entirely chemical based approach. Moreover, it can be applied in targeted areas where larval control could be impractical from agro-ecological perspective. The efficacy of screening doors, windows and eves on mosquito entry is well documented and it showed significant reduction in malaria vector mosquitoes [6, 9, 18, 22]. Study conducted in southern Ethiopia, indicated that screening materials fixed to doors, windows and eves lasted intact for at least 1 year which is double the age of longest IRS life span [23]. Thus, we propose to investigate the combined effect of LLINs and HS and LLINs, HS and PPT on entomological and epidemiological parameters.

The benefits of HS and PPT have been separately documented by several studies [8, 22–27]. Despite the evidence on the documented benefits, adoption of PPT and HS is quite limited. While PPT is adopted by only 260,000 farmers in sub-Saharan Africa [28], HS is still at the experimental stage [14, 29, 30]. The low adoption of these technologies necessitates not only introducing them in places where they are needed, but also evaluating their impact on health and livelihoods of the adopters of the technologies. How we best promote the technologies in the current study is also particularly interesting and will help draw lessons for the rest of Africa where the agricultural and health extension systems follow mostly a top-down approach of reaching farmers rather than being participatory [31].

### Trial objectives

#### General objective

The general objective of this intervention is to determine whether the combined intervention of long-lasting insecticidal nets, house screening and push-pull technology provide better protection against malaria and improves agricultural productivity in the intervention households compared to control households who received LLINs alone in Jabi Tehnan area, Northwest Ethiopia.

#### Specific objectives

i. To determine whether adding house screening of windows and doors of houses and implementing PPT reduces the rate of malaria parasite infection, parasite density, and anemia in children (aged between 5 to 14) compared to situations where only LLINs are used.
ii. To assess whether the proposed interventions reduce human mosquito-interaction (human biting rates, mosquito resting density, longevity, sporozoite rates, and the entomological inoculation rate (EIR)) inside houses compared with LLINs alone.
iii. To determine the incremental costs, benefits, and cost-effectiveness of adding house screening and PPT to usage of LLINs.
iv. To assess the economic, social, and environmental feasibility of the combined intervention of house screening, LLINs and PPT.

## Methods

### Study setting and period

This study will take place between September 2020 and December 2022 in the Jabi-Tehnan district of Amhara regional state, Northwest Ethiopia. The capital town is Finote-Selam, which is about 387 km from the National capital, Addis Ababa, and 176 km Southwest of the regional capital, Bahir Dar. The population of the district was 211,516 in 2017 with an average annual growth rate of 2.8% [32]. The district is divided into 38 villages *(Kebeles),* which is the lowest administrative unit of the country, and three town administrations. More than 90% of the people in the district live in rural areas practicing mixed farming.

The altitude of the district ranges from 900 to 2300 m above sea level. Much of the area lies in the higher altitude range, closer to 2300 m. Agro-ecologically, 88% of the district is classified as mid land and the remaining 12% as low land. The topography of the district is dominated by areas of flat plain. According to Asmare & Gure, 2019 [33], the topography is classified as 65% flat plain, 15% mountainous, 15% undulating and 5% valley. The rainfall distribution is uni-modal, and the rainy season normally lasts for 4 months from mid-May to mid-September. The annual rainfall ranges 1250 mm per annum. The mean minimum and maximum temperatures are 14 °C and 32 °C, respectively [33]. According to the report compiled from 17 villages *(Kebeles)* of the district, malaria is a major health problem threat resulting in total mortality of 4345, and average Plasmodium annual parasite rate (API) of 11% (Tsehaye 2018, personal communication). A cross sectional active malaria prevalence survey from randomly selected kebeles of the district in 2013 showed the disease prevalence of 2.8% [34]. According to the preliminary information collected from the district, houses are made of walls plastered with mud and roofs covered with corrugated iron sheet. According to the baseline survey conducted in the area 59% of the respondents reported that they keep the livestock in the same house but in separate rooms [35].

### Study participants

#### Population

The source population of this study will be the total population of the Jabi Tehnan district, Amhara Regional State, Northwest Ethiopia.

### Eligibility criteria

The study population will be all households that are found in the rural areas of Jabi-Tehnan district having at least one child (under 14 age) in their family.

### Sample size rationale

#### Sample size of houses for screening

Studies conducted in Ethiopia [12] and The Gambia [9, 10, 18, 36] have suggested that houses with their doors and windows screened can result in reduction of mosquito density measured between 40 to 70% as compared to control houses. In addition to mosquito proofing, house screening has proved to be effective intervention in reducing malaria incidence. Studies conducted to evaluate the impact of housing improvement on odds of malaria infection showed varying degree of efficacy. Tusting et al., [22], reviewed 53 published papers on housing and malaria interaction and reported 47% lower odds of malaria infection in ‘modern houses’ as compared to ‘traditional houses’. In Ethiopia, the protective efficacy of house screening was evaluated on 46 randomly selected households in Arba-Minch Zuria for 6 months and 61% reduction of malaria case was documented in screened houses as compared to the control houses [23]. In Kenya, malaria incidence was followed up in 80 screened houses for a year and 100% case reduction was reported [37]. Thus, considering the experiences from other countries and malaria prevalence information from the study area, this design is developed to measure the impact of house screening. A study conducted by Ayalew et al., (2016) [34] in 3 villages *(Kebeles)* in the district showed a 2.8% malaria prevalence and used as a benchmark for this trial. Accordingly, 838 households, will be selected from 30 villages *(Kebeles)*, using 80% power at the 5% significance level.

#### Sample size of houses for entomological data collection

According to WHO guideline for malaria entomology [38], a representative sample size for entomological collections per village will be 10 houses. Thus, a total of 40 houses (10 houses per treatment arm) will be used for mosquito collection. The treatment households are dispersed over three different geographical zones (low land, mid-land, and high land). Therefore, 40 households will be randomly selected for each geographical zone. Thus, a grand total of 120 houses will be selected for sampling mosquitoes from all three zones.

#### Sample size of households for social data collection

We will use the baseline data from the 3010 households collected in the district for the socioeconomic analysis. The sample is randomly selected from a fresh list of households in the rural and semi-urban part of the district. We will follow these households and collect additional information two times (baseline, midline, and end line surveys). The data collection procedure for the social data were discussed in detail in Abro et al. (2020) (doi.org/10.1257/rct.5642-1.0).

##### Recruitment

For this study, the principal investigator (AA) will be based in the study district and form a recruitment committee. The committee will be composed of the head of local administration, representative from village HEWs, the head of local health center and the principal investigator (AA). The team will make house-to-house visits and explain the objective of the study to the candidate household heads. The list of candidate households will be separately generated from the roster containing the list of household heads of the district by the data manager (ZA). In instances where the candidate household refuses to participate, reserve candidate households will be invited to participate.

### Design

#### Type of study design

A longitudinal study of four-armed household clustered randomized control trial will be conducted to estimate the incremental benefit of combining house screening, long-lasting insecticidal nets, and push-pull technology.

#### Study flow chart

**Fig. 1:**
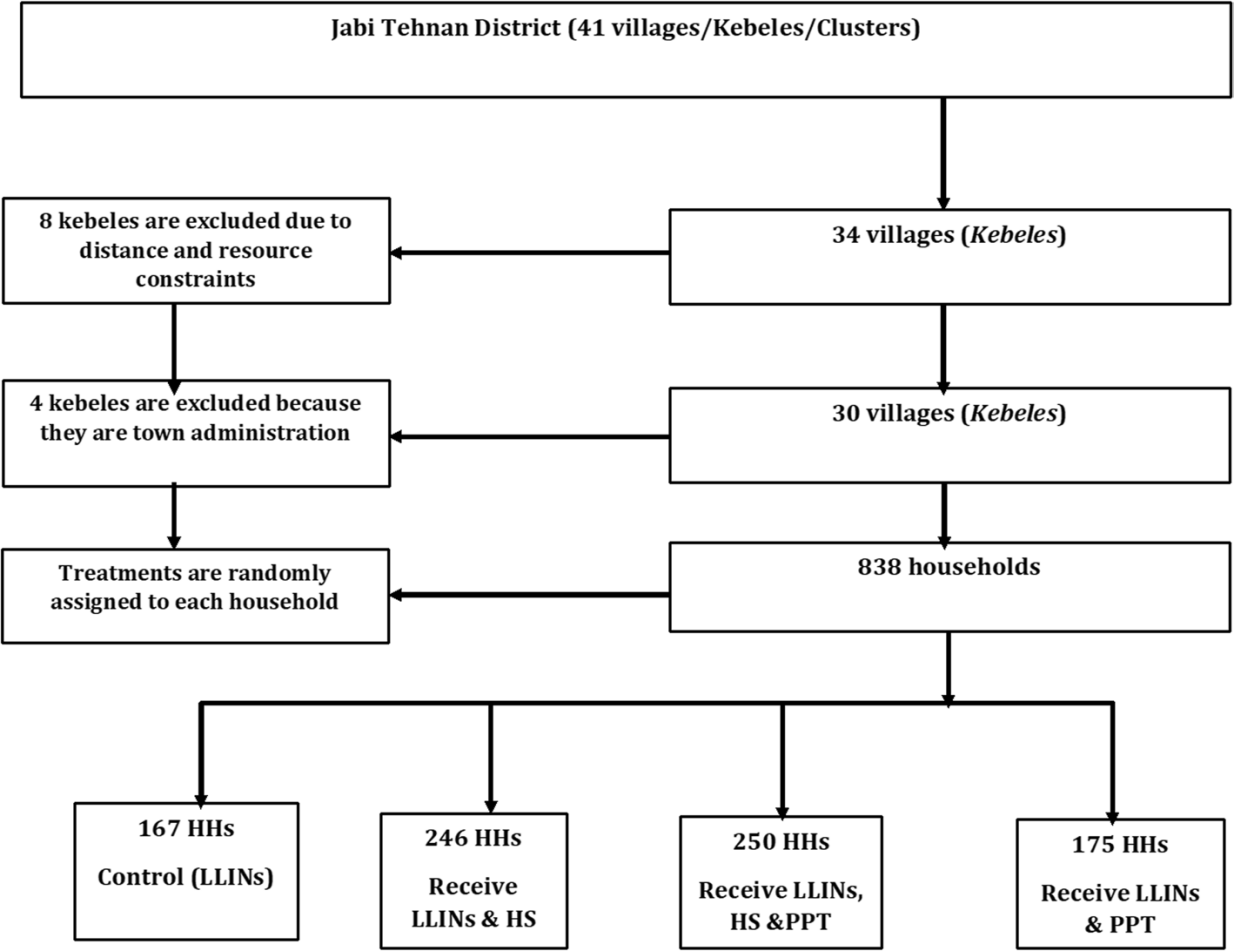
Flow chart of the study

The study design is summarized in a flow chart as shown in Fig. 1. The study contains four treatments randomly assigned to households with village as the blocking factor. As some houses get screening and others not, there could be a risk of diverting potentially infectious mosquitoes from screened houses to unscreened ones. Thus, to avoid such risks care will be taken to not exceed 5% of houses per village as it was recommended from previous studies from The Gambia [14]. Studies conducted in other countries confirmed that the risk of mosquito spread to unprotected houses is unlikely to increase if the proportion of houses protected is less than 10% [5, 14].

In this study, a total of 167, 246, 250 and 175 houses will be included in four different treatments, treatment 1 (control, LLINs only), treatment 2 (LLINs and HS), treatment 3 (LLINs, HS and PPT) and treatment 4 (LLINs and PPT), respectively. The differences in sample size across arms is due to differences in number of households within each sub-village. To full fill the total sample size, we did the random selection based on population size of each sub-village. Census of children under 14, and above 14 years of age will be made in all 838 households selected. One child will be randomly selected from each house for annual malaria parasite survey. In addition to the annual parasite screening survey, monthly follow up will be implemented in all households to monitor clinical malaria by health extension workers. If a child moves or quits the study, we will replace the child from the same house. In instances where the whole family leaves the area, we will document them as lost to follow up.

The selected children will be checked up every month for clinical symptoms of malaria infection, splenomegaly, and development of anaemia. Blood test will be conducted if the child develops clinical symptoms. The child will also be surveyed at the beginning and end of each transmission season to estimate the prevalence of *Plasmodium species* infection, parasite density, and the prevalence of anaemia. Collections will be made from both indoor and outdoor settings.

### Sequence generation

As described in the Section of study population all the households in the district are eligible to the treatment. The unit of randomization is the sub-village (*sub-kebele*) level. For this study, we divided the 30 villages *(Kebeles)* into 66 sub-villages/sub-kebeles. In each sub-kebele, we will obtain a fresh list of farmers, which are organized into one-to-five groups.^1^ Each *sub-kebele* will be randomly assigned into the four treatment arms.

### Randomization

As the study area is composed of different localities (and elevations) of the district, the risk of malaria infection also varies among *kebeles* (document review from district malaria information). Therefore, the study area will be first clustered into *kebeles* and then each kebele will be clustered in to *sub-kebeles*. The randomization process will be conducted using the STATA’s *sampsi* package and using *sub-kebele* as a unit of randomization. Efforts will be made to ensure that each *kebele* receives balanced number of treatments following the standard protocol developed by Pinder et al. (2016) [14]. Stratified randomization by *sub-kebeles* will take out the kebele effect and the likelihood of chance imbalances between study arms. All entomological, epidemiological, and social studies will be conducted following the same rule described above i.e., stratified randomization. The randomization will be done by ZA to prevent selection bias by concealing the allocation sequence from the field researchers assigning sub-kebeles into the four treatment arms until the moment of assignment. Thus, both the chief investigator and the principal investigator will not be involved in the randomization process.

### Blinding

Screening of windows and doors will not be blinded as it is difficult to conceal them. However, we will follow the World Health Organization 2015 [34] guideline in blinding other activities. The blood films will be read by microscopists blinded to the identity and intervention status of the subjects. Mosquito collection will be made by using standard CDC light traps thereby avoiding the potential bias that could be introduced by the fieldworkers to collect specimens. Mosquito identification will be made by different technicians who will not know the trap location.

### Interventions

#### Long-lasting insecticidal nets

DuraNet® (Shobikaa Impex pvt Ltd., Karur, Tamil Nadu 639,006, India) will be provided to all households (except control arm) at the rate of one bed net for two people following the NMCP and as per WHO recommended universal net coverage [1, 39]. The nets will be provided by NMCP district office and will be distributed to study households in the first week of July 2020. All study participants will receive new LLINs free of charge at the beginning of the intervention regardless of the previous ownership, with householders maintaining their existing nets at the time of distribution.

#### House screening

The house screening work will be undertaken by pre-trained artisans of about 4 to 5 people to be recruited from the study area. The training will be done by an in-country contractor familiar with screening of houses. The screening of 500 houses is expected to take about 2 months. Household owners will be trained on the care needed to keep the screens intact and effective and avoid activities that could result into making holes in the mesh or cause the screen to slide and create spaces that could allow mosquito entries into the houses. Other routine procedures to be used to reduce mosquito entry into houses such as closing windows and doors early will be emphasized and adherence to this practice monitored passively by the study team. As part of the intervention all the windows, doors and eaves will be screened using polyethylene material (POLYTEX INTERNATIONAL (UK) LIMITED, 14 Rutherford Way, Drayton Fields, Daventry, Northants, NN11 8XW, United Kingdom).

#### Push-pull technology

Push pull technology is a biological method of controlling pests of cereals: stem borer and striga. It is a novel pest control method in which a repellent intercrop and attractant trap plant is used simultaneously. It has three components which include the push, the pull, and the intercropped plant. The push component refers to desmodium plant, a.k.a. *Desmodium uncinatum*. The pull component refers to the brachiaria grass, *Brachiaria cv mulato,* which is usually planted at the periphery of the plot and the intercropped plant which is our target plant to be protected (Maize or Sorghum). While the desmodium is rich in protein, the brachiaria is rich in carbohydrate, which will serve as an important source of animal feed. The detail application of PPT is described elsewhere [40]. The desmodium plant releases a semiochemical that repels stemborer moths (push), and attracts their natural enemies, while brachiaria grass attracts them (pull). In addition to repelling the stem borer pests, desmodium is very effective in suppressing striga weed while improving soil fertility through nitrogen fixation and improved organic matter content. On the other hand, both plants provide high-value animal fodder, contributing to improved milk production and keep the health of the livestock [28, 41]. Both plants are proved to be drought resistant and can be used as source of animal feed in areas where rain is limited. According to a standard protocol developed by ICIPE for PPT application, a plot of any size can be used to implement the technology. A typical PPT plot is usually 25 by 25-m size. The brachiaria plants are sowed in three rows around the perimeter of the plot and separated by 75 cm width each other. The desmodium plant is sowed in alternate manner with the crop plant (maize or sorghum) [28].

Thus, as part of the intervention training will be provided to selected farmers on the concept of PPT and how to use it. These include plot preparation, sowing, establishing, managing, animal feed preparation and seed harvesting. A total of 425 farmers who successfully completed the training and who been selected to house screening treatment will be provided PPT plant seeds namely Mulato II Hybrid Brachiaria, (3 kg/ha, Barenbrug, 26 Prosperity Way Dandenong South VIC 3175, Australia) and *Desmodium intortum* 3-5 kg/Ha from the same company. The amount of Kg provided to each farmer will be decided on the size of plot made ready by the farmer.

#### Risks and harms

There is no risk of any infection as we do this study, however, finger pricking for blood test is usually with mild pain and discomfort.

### Data collection and management

#### Data collection methods

For this study, each household will be provided with a specific identification (ID) number. The child included in the study from each household will receive a unique personal, three-digit ID number (village number/household number/person number). All forms and datasets will identify participants by their unique identifier numbers, and names will not be used. The geographic coordinates of all study households will be documented at the beginning of the study and used in mapping the study points. Questionnaires and forms used will be initially prepared in English and translated into Amharic for data collection and then it will be translated back to English later. The questionnaire has already been validated. We will use the questionnaire for collection of the malaria KAP, socio-economic, socio-demographic, the acceptance of HS, challenges related to HS utilization, LLINs ownership and utilization. There are questions pertaining to PPT acceptance, utilization, and other opinion. Databases will be password protected and accessible only to authorized personnel. All documents will be securely stored in locked filing cabinets and accessible only to authorized personnel.

### Community sensitization and communication

Awareness creation on the purpose of the study, on the type of the interventions, and the need to involve in the study will be communicated to the community members through existing channels such as local government centres and through health extension workers. Farmers will be randomly selected to be part of the trial, and this could create some confusion among community members and lead into unnecessary misinformation. In this regard, the randomization process will be clarified to the community members to clear the misinformation and further explain that the community members at the receiving end of the intervention do not have any role whatsoever in the process of selection.

### Epidemiological data collection methods

A rapid diagnostic test (RDT), Microscopy and dry blood spot (DBS) will be used to measure the epidemiological parameters. In addition, prevalence of anaemia and splenomegaly among the study participants (children) will be graded according to their Hb level and by observation respectively. The axillary temperature will be taken fortnightly by health extension workers from all enrolled study children and if the child shows ≥37.5 °C or history of fever in the past 48 h, then, a rapid diagnostic test (RDT, CareStart® Malaria Pf/Pv combo test; Access Bio, Inc., Somerset, NJ, USA) will be conducted to further cross check the presence of malaria parasite. In line with this, blood spot sample (DBS) will be collected for later testing using PCR-based methods. In this regard we propose to collect two set of malaria case data. The first one is monthly epidemiological data that is collected based on clinical symptoms during the house-to-house visit and the second set of epidemiological data is obtained through active annual malaria prevalence survey. Monthly case follows up and testing of febrile cases will be conducted using malaria RDT kit whereas the annual active parasite screening will be done using diagnostic tools, Microscopy and DBS. The combination of diagnostics will be used to fill the gap among the tools. For instance, asymptomatic and sub-microscopic cases will be detected using the DBS PCR approach. The monthly house to house follows up will be made by health extension workers. The annual prevalence survey will be made by licensed laboratory technicians and health extension workers. In all instances, malaria positive children will be provided with a full dose of *Artemether-lumefantrine* (AL) and chloroquine by a trained health worker following national guidelines. In Ethiopia, CHWs are trained and mandated to test and treat malaria.

### Entomological data collection methods

A total of 10 houses, (five houses for indoor and five houses for outdoor LTs) catches will be selected from each study village. Indoor and outdoor catches will be collected from each study arm. Mosquitoes will be collected indoor and outdoor from 6:00 pm to 06:00 am from each selected house using standard battery-operated CDC light traps. Traps will be hung from the ceiling or from roof support at the foot end of the bed where people sleep at night and each trap will be suspended about 1.5 m from the floor. Traps will be also hung outdoor under the eaves of the house for outdoor mosquito collection. Each trap will be set by trained research team members. Collection bags will be retrieved from traps in each house in the morning between 06:00 am and 07:00 am. All unfed, fed, half gravid and gravid adult female *Anopheles* mosquitoes (Indoor and Outdoor) will be identified using taxonomic keys of Gilles and Coetzee (1987) [42]. Dried head and thorax of *Anopheles gambiae* s.l., and *Anopheles pharoensis* collected by CDC light traps from each study villages *(Kebeles)* will be carefully separated from the abdomen and tested for *P. falciparum* and, *P. vivax-210 and P. vivax-247* circumsporozoite proteins (CSPs) simultaneously following the protocol developed by Beier et al. 1988 [43]. On agricultural pest assessment, the severity of infestation will be scored on visual observation of the foliar damage attributed to each pest using a 1 to 5 scale, where 1 is clean with no visual infestation symptoms, 2 = very little damage, 3 = high level of damage where plants show the presence of FAW larvae feeding and most of the young leaves show infestation symptom, 4 = severe damage where almost 75% of the leaves are severely affected and excrement is visible on the infested areas and the maize whorls, and 5 = very severe damage where total plant damage due to FAW is visible.

### Socio-economic data collection methods

Semi-structured questionnaire will be prepared to collect socio-demographic data at the beginning and end of the study. Each study household will be observed for durability of the screening material, the presence of holes, tears, losses and the fitness of windows and doors. The presence or absence of LLINs and the number and age of people who slept under bed nets in the previous night will be assessed in every visit to mosquito sampling (before and after screening). The impact of screening intervention on bed net use rate will be measured by observing bed net use among household members before and after house screening intervention, and between intervention and control groups. For the social science component of this impact evaluation, the team will collect data using household surveys, focus group discussions and key informant interviews. A baseline survey, midline survey and end line surveys will be undertaken using a structured household survey questionnaire and trained enumerators.

### Study endpoints/outcomes

#### Clinical evaluations

The main outcomes of the clinical study will be the malaria incidence, splenomegaly, and anaemia. Thus, the baseline clinical survey of all study children will take place between Sept and Oct 2020 to determine the clinical parameters. As part of the follow up, starting from Nov 2020, trained health extension workers will visit the house of each child and take data of clinical symptoms, temperature, and splenomegaly (sign of spleen enlargement). While monthly follow up of the child continues throughout the 2 years of study period (Sept 2020 to Dec 2022) for documentation of any malaria incidence, blood testing of the entire cohort of the study children will be repeated at the end of each rainy season (Sept/Oct 2021) and Sept/Oct 2022). In between the annual clinical surveys (i.e., at the time of monthly follow up), blood testing will be done only to children who develop fever to minimize community fatigue development. Temperature measurement, RDT testing, blood haemoglobin measurement, blood sample collection for microscopy will be conducted by a licensed laboratory professional following the WHO guideline [44].

### Entomological evaluations (medical entomology)

The major entomological outcomes of the study are changes in mosquito densities, rates of mosquito infection (sporozoite rates), and the entomological inoculation rate (EIR). Both indoor and outdoor mosquito collections will be made using the Centres for Disease Control (CDC) light traps to estimate the mosquito density. Collections will be made twice per year (July to Sept and January to March) starting from July 2020 to Oct 2022. All mosquito collections will be preserved in silica gel and transported to the International Centre of Insect Physiology and Ecology (ICIPE) Kenya, Nairobi, ICIPE molecular laboratory, where they will be identified to species level and examined for sporozoite infection using Polymerase chain reaction (PCR) technique. Blood meal analysis of fed females will be done using Enzyme linked immunosorbent assays (ELISA).

### Entomological evaluations (agricultural entomology)

In line with the investigation of vectors of medical entomology, the agronomics team will evaluate the efficacy of PPT against stem borers, fall armyworm (FAW) and striga infestation. Visual observation will be made in selected plots from each treatment arm (PPT plot and control) and the degree of infestation will be measured using scale scores where 1 attributes to clean plant with no infestation and 5 attributes to very severely damaged plant [45].

### Economic and social science evaluations

The actual outcomes for the economic and social evaluations are discussed in detail in Abro et al. 2020. “Social networks, incentives, and diffusion of house screening and push-pull technology interventions in rural Ethiopia.” AEA RCT Registry. April 06. 10.1257/rct.5642-1.0. The primary outcomes are PPT knowledge score, PPT and HS adoption productivity of maize (kg/ha), milk productivity (kg/animal), cost of illness (USD/household), malaria prevalence (%), lost working days due to malaria, and lost school days due to malaria for children. We have also other secondary outcomes such as willingness to pay for PPT and HS, women and children dietary diversity score, and household food insecurity access scale (HFIAS) [46].

### Data management and access

Data will be stored in two forms, i.e., hard, and soft copies in compliance with the principles of good clinical practice protecting the confidentiality of participants. Specimens of mosquitoes, blood film slides and genotype print of PCR outcomes will be maintained by the principal investigator and be available up on request from authorized representatives, regulatory bodies. The results of the study will be made publicly available through peer reviewed journals.

### Statistical methods

#### Malaria incidence data analysis

Change in clinical malaria incidence over 2 years period among the treatment and control arms will be determined using Poisson distribution. In this trial we will follow modified ITT protocol and if a child is lost from follow up, or withdraw from the study, or refuse to be included in the treatment, it will be immediately replaced by a child from the reserve list. The Relative Risk (RR) and RR Reductions (RRR) will be calculated with corresponding 95% confidence intervals to compare dichotomous variables, and difference in means will be used for additional analysis of continuous variables. Mixed effects Poisson model will be used to test the difference in incidence rates among the study arms, to determine effects of the repeated measurements within house, village, the effect of year and village-intervention interaction effects. To control the effect of clustering or village and individual level confounding factors such as gender and age, these covariates will be fitted in to random effects during analysis. If a child is diagnosed with malaria case within 28 days of the first episode with the same plasmodium species, then it will be put on treatment as part of the safety protocol, however, the case will not be included in the analysis. The prevalence of anaemia among the study participants (children) will be analysed using the guideline developed by world health organization [47]. Accordingly, all children between 6 and 59 months of age, whose Hb level is recorded between 10.0–10.9 g/dl, 8.0–9.9 g/dl and less than 8.0 g/dl will be graded as mild, moderate, and severe, respectively. In line with this we will also report community level anaemia prevalence. Thus, a prevalence of anaemia will be stated as severe if it is documented over 40% (combining mild, moderate, and severe) and moderate if the prevalence is 20–39.9%. Both malaria and anaemia prevalence data will be compared in the intervention and control houses using multilevel mixed-effects logistic regression models, taking village effects into account.

### Entomological data analysis

Indoor and outdoor *Anopheles* densities will be compared for each study arm using a student t-test. Overall mosquito density among treatment arms will be compared using one-way analysis of variance (ANOVA) and if there is significance difference among treatment arms mean separation test will be done using turkey’s range test. The sporozoite rate will be determined as the proportion of malaria vectors positive for CSPs over the total number tested for CSPs.

The durability test of screening material will be conducted following the guideline developed by Kinde et al. (2018) [23] and modification of world health organization [48] guideline for evaluation of durability of long-lasting insecticidal mosquito nets under operational conditions. Accordingly, the durability of the screening intervention will be measured by assessing the number of holes on the meshes. Fabric integrity of all HSs fixed into windows and doors will be assessed for holes at each monitoring round. The proportion of HSs with any holes will be presented with total number of HSs in surveyed households as denominator. The scale of HS damage will be quantified using hole index formula recommended for mosquito nets and it is given as Hole index = (A x no. of size-1 holes) + (B x no. of size-2 holes) + (C x no. of size-3 holes) + (D x no. size-4 holes). Numbers 1, 2, 3, 4 refer to the size of the whole, letters A, B, C, D refer to the weight of the hole and given as 1, 23, 196 and 578 [48]. The fabric integrity of screens fixed in doors will be analysed separately as its exposure to wear and tear is higher as compared to that of windows. In depth interviews, photo-based observation will be conducted to measure community acceptance of the intervention.

### Social science data analysis

The socioeconomic data analysis will be done using descriptive and econometric approaches. An incremental cost effectiveness ratio (ICER) will be calculated for each outcome and arm using standard Disability Adjusted Life years (DALYs). The interventions will be ranked according to cost effectiveness, whereas inequality in terms of health outcomes, will be measured by the Gini coefficient and the concentration index.

#### Handling of dropouts/withdrawals

The right of participants to withdraw from the study at any time without giving a reason will be communicated prior to recruiting each participant. If the first child from the house is moved to other place for various reasons, the second child will be replaced. In unlikely scenario of the situation in which the entire family moves (e.g., building home in nearby town is a common practice in the area), the follow up will be discontinued and other related data collection activities (entomology and social science) will be replaced by reserve house.

### Safety and monitoring

#### Safety evaluations

House to house visit, children follow up and RDT testing during monthly follow up period will be conducted by the trained health extension workers (HEWs). Yearly blood sample collection and processing will be handled by professional. Blood samples for RDTs, microscopic examination and Haemoglobin test measurements will be collected using aseptically disposable lancets. Positive, cases found during the monthly follow up will be treated by the HEWs according to the national guidelines [40] and any complications that could potentially develop in to series adverse effect (SAE) on participants will be referred to the nearby health center or hospital and reported to the principal investigator.

### Trial oversight

The implementation of this clinical trial will be overseen by Amhara regional public health institute (partner and legal body which ensures the proper implementation of ethical protocols) and NORAD, sponsoring the trial. A contract agreement will be signed among the three parties (the PIs, the sponsors, and the regulatory partners). As part of the contract the PIs will submit annual reports detailing the progress of the trial, the safety procedures put in place and the overall impact of the study. Blood sample collection and treatments are part of routine malaria control in Ethiopia and will be undertaken in collaboration with the health workers at the health posts and therefore, no need to oversee the routines. Side wastes that result from the trial such as used insecticide nets, empty sachets, cartons, plastic bags, used gloves, pricking needles and other contaminated materials will be properly disposed following the guideline set by Robertson et al. (1995) [49].

### Ethics approval

The study was approved by the IRB of the Amhara public health Institute, Amhara reginal state (ref: APHI/HRTTD/03/341/2019) and renewed up on evaluating the trial progress with ref.: H/R/T/T/D/5/3 and date 20/08/2021. The protocol was registered online on Date 28/05/2020 on site **www.pactr.org** With registration No: **PACTR202006878245287**. The copy of ethics approval letter is prepared in separate document (Additional file 1).

### Informed consent

Informed consent will be obtained from each study participant. In case of children enrolled into the study package the family or guardian will be consulted to get their oral and written consent. In addition to consent from family/guardian, informed consent will be sought for children above 12 years old. For illiterate participants the purpose and detail content of the consent form will be read by the PI and their consent to participate in the study will be requested. Detail consent form which includes the title, the purpose, intervention procedure, benefits, risks, benefits, right to refuse and to discontinue, and PI contact information are prepared in separate document (Additional file 2).

### Confidentiality

Blood slides taken from a child will not be used for other purposes other than described above and the records in which the subject is identified will be maintained confidential and blood slides collected will not be accessed by other parties other than the investigators and at the end of the study slides will be safely disposed. Names of participants will be filled in confidential logbook. Codes, not names of participants will be used to label Blood film specimens. The names of the study participants will be accessed by the PI only for trace back participants who were malaria positive.

### Dissemination policy

Three scientific papers will be drafted and published on internationally recognized peer reviewed journals. These include 1) the combined effect of HS and PPT on improving the human health with specific focus on clinical malaria, 2) the combined effect of HS and PPT on improving animal health and maize crop production 3) the cost of malaria burden and cost effectiveness of house screening as reflected by the end-users. In addition to the publications planned stakeholder workshop will be conducted in the district where the project is being implemented with objective disseminating the major findings of the project.

Timelines of activities

**Table:** 

**Activity by year and month**	**J**	**F**	**M**	**A**	**M**	**J**	**J**	**A**	**S**	**O**	**N**	**D**
2019												
Project submission to IRB and approval	X	X	X	X								
Development of data collection tools					X							
Sensitization of study population												
Census and mapping						X	X					
Epidemiological and entomological pilot study							X	X	X	X	X	X
2020												
Selection of study villages *(Kebeles)*	X	X										
Randomization			X									
Procurement of screening material				X	X							
Procurement of Desmodium & Brachiaria seed				X	X							
Seed distribution and plantation						X	X					
Screening of selected houses					X	X	X					
Protocol registration								X	X	X		
Epidemiological & entomological Data collection	X	X	X	X	X	X	X	X	X	X	X	X
Social studies data collection								X	X	X	X	
Cost effectiveness data collection								X	X	X	X	X
2021												
Malaria prevalence survey					X	X	X	X	X	X	X	X
Entomological Data collection	X	X	X	X	X	X	X	X	X	X	X	
Entomology lab assays											X	X
Annual social studies survey								X	X	X	X	
Data entry and cleaning	X	X	X	X	X	X	X	X	X	X	X	X
Cost effectiveness study								X	X	X	X	X
2022												
Malaria prevalence survey					X	X	X	X	X	X	X	
Entomological Data collection	X	X	X	X	X	X	X	X	X	X	X	
Entomology lab assays											X	X
Annual social studies survey					X	X	X	X	X	X	X	
Data entry and cleaning	X	X	X	X	X	X	X	X	X	X	X	
Cost effectiveness study					X	X	X	X	X	X	X	X
Dissemination of findings to the study community									X	X	X	X
Publication and final report to stake holders									X	X	X	X

## Discussion

Countries and inter-state actors who work in public health sectors are running out of options to contain vectors of malaria vectors as existing interventions become less effective due to growing insecticide resistance [4] and adaptive behavioral shift of vector mosquitoes to evade the interventions [50]. Thus, all options including research for novel vector control interventions [51], combination of existing strategies (e.g. combination of LLINs and IRS, [52] and supplementing them with auxiliary strategies e.g. combination of LLINs and HS, [14] are being tried as part of the ongoing research and innovation in the field of vector control. In addition, a recent modelling study conducted by Kassie et al. (2020) [53] showed that integrated interventions aimed at relaxing the constraints of multiple health problems (animal-human-plant health) are more effective in improving the overall welfare of humans as compared to the stand alone interventions. Thus, in this study we aim to test and assess the combined effect of 1) LLINs and HS 2) LLINs, HS and PPT and 3) LLINs and PPT. Households that receive LLINs alone will be used as control.

Screening houses for the purpose of excluding mosquito vectors into residential structures is well established practice in Europe [54], America [55] and some parts of tropics [5] however, these experiences were given less emphasis in Africa as main stay vector control intervention (LLINs and IRS) predominated the vector control arena. A specific design of the house (e.g. thatched roof, Iron sheet cover, closed eve, open eve) can play critical role in increasing or decreasing the odds of malaria infection [9] and mosquito density per capita or per house [36, 56,  57]. In addition to alleviating malaria, the introduction of house screening intervention in houses has resulted in reduced infection of other vector borne diseases such as dengue, leishmaniasis, yellow fever and Zika [58].

Push pull technology on the other hand is a semiochemical based intervention in which two plant species are being used in integrated approach with primary objective of increasing the quality and quantity of crop yield per unit area. It is developed at ICIPE research center, Kenya, in collaboration with Rothamsted research center UK and scaled up throughout Africa to support smallholder farmers [59]. The strategy is non-synthetic chemical, and it involves the intercropping of cereal crops with a forage legume, desmodium, and planting Napier grass as a border crop [27]. In this approach the *Desmodium intortum* plant releases the semiochemical which repels stem borer moths, and attracts their natural enemies, while Napier grass (now replaced by *Bracharia mulatotu*-II) attracts the pest. In addition, the *Desmodium* plant can effectively suppress striga weeds and improve soil fertility through nitrogen fixation. Both plants provide animal fodder thereby increasing the health and productivity of livestock. Commercialization of these companion crops can be used as a source of income generation through seed production. By integrating the push-pull technology and malaria vector control interventions, we hypothesize that the PPT will improve the livelihood of farmers through increased production and productivity thereby the synergy of the technologies contributing to improved overall life standard including the fight against malaria.

We expect some mild risks arising from this study. Screening of windows and doors may lead to reduced aeration in houses which may result in respiratory infection of children as it was hypothesized in similar studies conducted in The Gambia [14]. Routine monitoring of respiratory infections will be made on households enrolled in the study to document if there exists any incidence. In case of encountering any such infections, children will be referred to nearby health center for immediate treatment. We hypothesize that there would be significant reduction in mosquitoes entering houses due to screening which may lead to complacence in using bed nets. As part of routine monitoring and house-to-house visit, we will make sure that people continue to use LLINs as usual and educate them that HS is not a stand-alone intervention.

People involved in this study will benefit from freely available house screening materials and push pull technology. Their houses (windows and doors) will be screened, and they will be trained on how to use the push pull technology. As part of the technology transfer, each farmer will be provided with training and seeds of *Desmodium* and *Bracharia*. Agricultural development agents (DAs) will visit each household regularly to support the establishment of the technology on their farm. In addition, children enrolled in this study will benefit from regular check-ups and treatment whenever there is malaria incidence.

## Conclusion

This trial will provide critical information on whether combining LLINs, HS and PPT will have additive value in malaria control efforts. This study will also generate useful information on cost effectiveness of the intervention in combination and when applied singly. In addition, the acceptability of both HS and PPT interventions by the community will be carefully assessed. Thus, the findings of this study will be used for an effective planning and implementation of vector control interventions by national malaria control programs.

## Supplementary Information

**Additional file 1.** Ethics Review approval letter.

**Additional file 2.** Ethics information sheet and consent form.

## Data Availability

The datasets used and/or analysed during the current study are available from the corresponding author on reasonable request. All data generated or analysed during this study are included in this article and its supplementary information files.

## Abbreviations

CDC: Centers for Disease Control and Prevention
CV: Coefficient of variation
EIR: Entomological inoculation rate
FAW: Fall Armyworm, Hb, hemoglobin
LLIN: Long-lasting insecticidal nets
PCR: Polymerase chain reaction
PI: Principal investigator
RDT: Rapid diagnostic test
WHO: World Health Organization

## Footnotes

1 The one-to-five groups are the lowest units of farmers’ organizations that five farmers and a chairperson organize themselves into a group. As of April 2019, The Jabi-tehnan district had a total of 5608 one-to-five-groups of farmers.
