# Supplementary material for: The combined impact of LLINs, house screening, and pull-push technology for improved malaria control and livelihoods in rural Ethiopia: study protocol for household randomised controlled trial
Source: BMC Public Health. 2022 May 10;22:930. doi: 10.1186/s12889-022-12919-1 (PMC9088127; doi:10.1186/s12889-022-12919-1)
Supplement: Supplementary file 1 — Additional file 1. Ethics Review approval letter. [file 12889_2022_12919_MOESM1_ESM.docx]

**International center for Insect Physiology and Ecology (*icipe*)**

**Addis Ababa, ICIPE**

**Integrated vector management unit**

**Consent Form for field data collection**

**Part A: Information Sheet**

***Name of the Principal Investigator****: Abebe Asale* (Dr.)

***Name of the organization****:* **International center for Insect Physiology and Ecology (*icipe*)**

**Name of Sponsor: THE NORWEGIAN DEVELOPMENT AGENCY (NORAD)**

**Prepared by:** This consent form sheet is prepared by *Dr. Abebe Asale* whose main aim is to study *The combined impact of LLINs, house screening pull-push for improved malaria control and livelihoods in rural Ethiopia: study protocol for household randomised controlled trial.* The investigator is a staff member in **International center for Insect Physiology and Ecology (*icipe*)**

**Background**

Malaria prevalence in Ethiopia is generally considered as low. The 2015 MIS data reported that parasite prevalence in Ethiopia was 0.5% by microscopy and 1.2% by RDTs for areas below 2,000 meters and less than 0.1% prevalence in areas 2,000 meters above sea level. *Plasmodium falciparum* constituted 77% of infections detected below 2,000 meters (EPHI, 2015). According to the malaria risk map of the country, areas with the highest malaria transmission risk as stratified by district Annual Parasite Incidence (API) appear to be largely in the lowlands and midlands of the western border with South Sudan and Sudan. Many densely populated highland areas were newly classified as malaria-free (API=0), including the capital city of Addis Ababa.

In terms of malaria disease problem, Amhara regional state stands among the most burdened in malaria prevalence, after Gambella (18.4%), Benisahngul (10.4%) and Tigray (1.9%). Malaria prevalence in Amhara region is 1.1% and is close to the country’s average of 1.2% (EPHI, 2015). Approximately 75%t of Amhara’s population lives in malarious areas and 80% of the region’s landmass is favourable for malaria transmission, with malaria primarily associated with altitude, rainfall and development projects. Like other regions, the peak of malaria incidence follows the main rainfall season (July to September) and short rainy season (March to April/May). Consequently, malaria transmission tends to be highly heterogeneous geo-spatially within each year as well as between years. In 2016/2017, the total number of laboratory-confirmed plus clinical malaria cases were 158,812 with 80% of these cases being reported from zones harbouring development project (Tsehay, 2018; personal communication).

*In Jabi Tehnan district* malaria disease continued to be major health threat resulting in total morbidity of 4,345, and average annual parasite rate (API) of 11.23. A cross sectional active malaria prevalence survey from randomly selected kebeles of the district in 2016 showed the disease prevalence of 2.8% (Ayalew et al., 2016).

Therefore, the objective of this survey is to test and assess the combined effect of 1) LLINs and HS 2) LLINs, HS and PPT and 3) LLINs and PPT. Households that receive LLINs alone will be used as control.

The research is highly relevant to develop appropriate preventive and intervention measures that help in reduction of disease burden, reduction of working hours lost due to malaria and school absenteeism related to malaria episodes. It provides evidence-based information for the ministry of health to revitalize the importance of integrated vector management strategy in NMCP.

***Procedure:***

We kindly invite you to take part in this project, which is aimed measuring the prevalence of malaria-related fever and malaria parasitemia among populations under the age of 14 in Jabi Tehnan districts. If you are willing to participate in this project, you need to understand and sign the agreement form.

**Site and Participant selection**

Selected head of the households who are living in the catchment area of the district and who are volunteer will participate in the research. For children to be part of the study sample, consent will be sought from their parent or guardians.

**Voluntary Participation**

Participation in this research will be entirely voluntary. It is your choice whether to participate or not. You can change your mind later and stop participating even if you agreed earlier.

**Type of research intervention**

This research will involve interviews, taking blood samples for smear by finger pricking.

**Procedures**

The study has two components: active surveillance and malaria prevalence. In the census survey, every house in each selected and mapped villages will be visited by the data collectors together with guiders and censused using a questionnaire to document individual and household characteristics. Hence, each heads of the household in all study villages will be interviewed using a semi - structured questionnaire.

In active surveillance, Blood smear will be taken for making thick and thin film from a child by a trained health workers using sterile lancet/pricker who will visit the school every months for a year by pricking his/her finger if he/she found febrile by the health workers. If the child is presented with fever/malaria during house to visit appropriate drugs will be prescribed within 24 hours of the microscopic diagnosis. Blood smears will be brought to nearby Health center Laboratory for detection and identification of malaria parasites by experienced technicians. Finger pricking will be done with great care to avoid unnecessary physical suffering and injury on child during skin piercing.

Proper preparations and safe handling of materials such as lancets/skin piercing materials will be made to avoid contamination or avoid transmission of microorganisms and this will be strictly supervised by the principal investigator and qualified and experienced physicians who are also co investigators of this study.

**Incentives:**

You will be not provided any specific incentive for taking part in the research other than acknowledgment.

**Confidentiality**

Blood slides taken from a child will not be used for other purposes other than described above and the records in which the subject is identified will be maintained confidential and blood slides collected will not be accessed by other parties other than the investigators and at the end of the study slides will be safely disposed.

**Benefits:**

As a parent of the child who participate in this study you will be benefited from freely available house screening materials and push pull technology. Your house (windows and doors) will be screened and you will be trained how to use the push pull technology. As part of the technology transfer you will be provided with training and seeds of Desmodium and Bracharia. If the blood film taken from your child is positive, the child will be treated with a standard dosage of anti- malarials and you will not be charged for the treatment. During the course of the research there will be consultation period which all the district leaders and village leaders will be invited to know about the progress of the research and this will be arranged twice a year.

**Right to refuse or withdraw:**

Participation is voluntary, refusal to participate will involve no penalty or loss of benefits to which the Parents/guardians or the child is otherwise entitled and the parents/guardians may discontinue participation of their child at any time without penalty or loss of benefits to which the subject is otherwise entitled and confidentiality of records identifying the subject will be maintained.

**Risk and Discomfort**

There is no risk of any infection as we do this study.

***Benefits***

Your participation will help us in assessing the combined effect of *long-lasting insecticidal nets, House screening and Push-Pull agricultural technology for malaria prevention in Ethiopia*, which will be an important information for policy decision making.

***Incentives***

You will not be provided any incentives to take part in this research.

***Confidentiality:***

The information that we collect from this research project will be kept confidential. Information about you that will be collected from the study will be stored in a file, which will not have your name on it, but a code number assigned to it. It will be kept under lock and key, and it will not be revealed to anyone except the principal investigator and the concerned health professional.

***Right to refuse or withdraw***

You have full right to refuse from participating in this research if you do not wish to participate or to quit at any time; and this will not compromise the health services you get at the health institutions in any way at any time.

***Whom to contact***

If you have any questions you can contact any of the following two individuals and you may ask at any time you want:

Principal Investigator: ***Dr. Abebe Asale*, *icipe***

Telephone no:+*251900462162*

*Prof. Clifford Mutero icipe, Kenya*

*Tel:+254704835177*

This study was conducted after getting approval from Amhara regional public health institute institutional review board.

**Part B: Certificate of consent for Informed Consent from volunteer participants**

I have been requested take part in the study of “*Combining long-lasting insecticidal nets, House screening and Push-Pull agricultural technology for malaria prevention in Ethiopia: Protocol for Randomized control trial*” and I have read the foregoing information, or it has been read to me. I have had the opportunity to ask questions about it and any questions I asked have been answered to my satisfaction. I consent voluntarily to collaborate in this study and understand that we have the right to refuse or to quit at any time without affecting our right.

Print Name of Subject Date and Signature or thumb impression of Subject

___________________________ _______________________

___/___/___ (dd/mm/yy)

If illiterate

Print Name of Independent Literate Witness Date and Signature of Witness (if possible, this person should be selected by the participant and should have no connection to the research team)

___________________________ ___________________________

___/___/___ (dd/mm/yy)

Print Name of Researcher/Moderator Date and Signature of

Researcher/Moderator

___________________________ ___________________________

___/___/___ (dd/mm/yy)
