# Supplementary material for: The combined impact of LLINs, house screening, and pull-push technology for improved malaria control and livelihoods in rural Ethiopia: study protocol for household randomised controlled trial
Source: BMC Public Health. 2022 May 10;22:930. doi: 10.1186/s12889-022-12919-1 (PMC9088127; doi:10.1186/s12889-022-12919-1)
Supplement: Supplementary file 2 — Additional file 2. Ethics information sheet and consent form. [file 12889_2022_12919_MOESM2_ESM.docx]

**International center for Insect Physiology and Ecology (*icipe*)**

**Addis Ababa, ICIPE**

# In this project we plan to collect blood samples in the form of dry blood spot (DBS) from selected households once per year for two years. Detail collection procedures described in the body of the documents. In addition, Mosquito samples will be collected from selected households. The efficacy and survivor rate of screening material and community acceptance will be assessed using pre-designed follow up questionnaire. All specimens (human blood samples and mosquito collections) will be collected in dry storage form. The samples will be stored in icipe, Addis Ababa, entomology laboratory. Only authorized people by the PI will have access to the sample for further processing.

# PART 1: Malaria examination report form

| **Patient details** | | |  | **Requester details:** | |
| --- | --- | --- | --- | --- | --- |
| Name: |  | |  | Name: |  |
| Address: |  | |  | Organization |  |
| Telephone number: |  | |  | Address: |  |
| Date of Birth: |  | |  | Telephone number: |  |
| Gender: | Male | Female |  |  |  |

**Sample details:**

| Urgency: | Normal |  | Sample taken from patient: | |
| --- | --- | --- | --- | --- |
|  | URGENT |  | Date: | (dd/mm/yyyy) |
|  |  |  | Time: | (hh/mm) |
| Fasting | Non-fasting |  |  | |

| Blood  Faeces | Urine  Sputum | Swab  Fluids | Tissue  Cytology |
| --- | --- | --- | --- |
| Other, namely: |  |  |  |
|  |  |  |  |

**Relevant clinical information:**

| Drug therapy: |  | Last dose: | |  |
| --- | --- | --- | --- | --- |
|  |  | Date: | (dd/mm/yyyy) | |
|  |  | Time: | (hh/mm) | |
| Other relevant clinical information: |  | | | |
|  |  | | | |
|  |  | | | |

**Examination requested:**

| **Profile test** | | **Biochemistry** | | **Hematology** | **Microbiology** | **Anatomical Pathology** |
| --- | --- | --- | --- | --- | --- | --- |
| G2000  G 2000-X  GT9  GTI  NEO  ES  HB3 | DFS  LFT  RFT  TFT  MAC  LGL  LIP | CEA  CA 1  CA 5  CA 9  PSA  AFP  Glucose | HIV 1 & 2  HbA1c  HBsAg  H. pylori  Uric Acid  Free T4 | FBE (incl. ESR)  FBC  Hb  TWDC  Platelets  ABO & Rh (D)  Malaria parasites | Urine FEME  RPR (VDRL)  Microscopy/Culture/Sensitivity  AFB (ZN) Smear Only  AFB Smear & Culture | Histology  Non-Gynae/FNA  Site:   \|  \| \| --- \| |

| **Additional tests:** | | | | **Cervical Cytology:** | | | | | | | |
| --- | --- | --- | --- | --- | --- | --- | --- | --- | --- | --- | --- |
|  | | | | Pap smear  Normal  Post-Mono Blood  Susp lesion | | | | | | | |
|  | | | |  |  |  |  |  |  |  |  |
|  | | | |  |  |  |  |  |  |  |  |
|  | | | |  |  |  |  |  |  |  |  |
|  | | | | Other: | | |  | | | |  |
|  | | | | Site | Cervix  Vault  Other, namely: | | | | | Endocx  Lat. Vag. Wall. | Post Fornix |
|  | | | |  |  |  |  |  |  |  |  |
|  | | | |  |  |  |  |  |  |  |  |
|  | | | | LMP | | (dd/mm/yyyy) | | | | |  |
|  | | | | Post – menopausal  HRT (hormone Replacement | | | | | | | |
|  | | | |  |  |  |  |  |  |  |  |
|  | | | | Other, namely: | | | |  | | | |
|  | | | |  | | | | | | | |
| **Date:** | **(dd/mm/yyyy)** |  | **Requester’s signature:** | | | | | |  | | |

**House screening follow up format**

**በርና መስኮት አጎበር የተገጠመላቸዉን ቤቶች የወባ በሽታ ሁኔታ መከታተያ ቅጽ**

ቀበሌ _____________ ጎጥ ____________፣አባወራ ሙሉ ስም ________________ ዓ.ም. ___________

| ወር | ቤት ዉስጥ በወባ ታማሚዎች ብዛት | ከ5ዓመተ በታች | 5 እስከ 14 | ከ14 በላይ | ነፍሰ-ጡር | ፋልስፓራም | ቫይቫክስ | ቅልቅል |
| --- | --- | --- | --- | --- | --- | --- | --- | --- |
| ጥር |  |  |  |  |  |  |  |  |
| የካቲት |  |  |  |  |  |  |  |  |
| መጋቢት |  |  |  |  |  |  |  |  |
| ሚያዚያ |  |  |  |  |  |  |  |  |
| ግንቦት |  |  |  |  |  |  |  |  |
| ሰኔ |  |  |  |  |  |  |  |  |
| ሐምሌ |  |  |  |  |  |  |  |  |
| ነሐሴ |  |  |  |  |  |  |  |  |
| መስከረም |  |  |  |  |  |  |  |  |
| ጥቅምት |  |  |  |  |  |  |  |  |
| ሕዳር |  |  |  |  |  |  |  |  |
| ታህሳስ |  |  |  |  |  |  |  |  |
| ጠቅላላ |  |  |  |  |  |  |  |  |

ከ ICIPE (ኢስፔ) የመስክ ጥናትና ዳታ ጠራት ተቆጣጣሪ (ስምና ፍርማ)

ስም፡ ______________________________

ፍርማ፡ ____________________________

ቀን፡ ______________________________

ከ ቀበሌዉ የጤና እክስቴንሽን መረጃ ሰብሳቢ (ስምና ፍርማ)

ስም፡ ______________________________

ፍርማ፡ ____________________________

ቀን፡ ______________________________
